# Influence of the Al-Doped ZnO Sputter-Deposition Temperature on Cu(In,Ga)Se_2_ Solar Cell Performance

**DOI:** 10.3390/nano12193326

**Published:** 2022-09-24

**Authors:** Hyeonwook Park, Salh Alhammadi, Vasudeva Reddy Minnam Reddy, Chinho Park, Woo Kyoung Kim

**Affiliations:** 1 Korea Institute of Energy Technology (KENTECH), Naju 58330, Jeollanam-do, Korea; 2 School of Chemical Engineering, Yeungnam University, Gyeongsan 38541, Gyeongbuk, Korea

**Keywords:** Cu(In,Ga)Se_2_, surface roughness, CIGS, AZO, transparent conductive oxide, TCO, solar cells

## Abstract

Heterojunction Cu(In,Ga)Se_2_ (CIGS) solar cells comprise a substrate/Mo/CIGS/CdS/i-ZnO/ZnO:Al. Here, Al-doped zinc oxide (AZO) films were deposited by magnetron sputtering, and the substrate temperature was optimized for CIGS solar cells with two types of CIGS light absorbers with different material properties fabricated by three-stage co-evaporation and two-step metallization followed by sulfurization after selenization (SAS). The microstructure and optoelectronic properties of the AZO thin films fabricated at different substrate temperatures (150–550 °C) were analyzed along with their effects on the CIGS solar cell performance. X-ray diffraction results confirmed that all the deposited AZO films have a hexagonal wurtzite crystal structure regardless of substrate temperature. The optical and electrical properties of the AZO films improved significantly with increasing substrate temperature. Photovoltaic performances of the two types of CIGS solar cells were influenced by changes in the AZO substrate temperature. For the three-stage co-evaporated CIGS cell, as the sputter-deposition temperature of the AZO layer was raised from 150 °C to 550 °C, the efficiencies of CIGS devices decreased monotonically, which suggests the optimum AZO deposition temperature is 150 °C. In contrast, the cell efficiency of CIGS devices fabricated using the two-step SAS-processed CIGS absorbers improved with increasing the AZO deposition temperature from 150 to 350 °C. However, the rise in AZO deposition temperature to 550 °C decreased the cell efficiency, indicating that the optimum AZO deposition temperature was 350 °C. The findings of this study provide insights for the efficient fabrication of CIGS solar cells considering the correlation between CIGS absorber characteristics and AZO layer deposition temperature.

## 1. Introduction

The significant increase in energy demand in recent years owing to rapid industrial and population growth has resulted in heavy dependence on non-renewable energy sources, causing serious environmental issues. Solar energy has emerged as a promising renewable energy source owing to its unlimited supply and non-polluting nature. Among the various types of solar cells, such as wafer-based crystalline Si and thin-film solar cells, Cu(In,Ga)Se_2_ (CIGS) solar cells exhibit superior performance compared to other photovoltaic technologies, including a high efficiency (23.35%) [1], outstanding long-term stability, and the applicability of growth on a flexible substrate [2]. Consequently, CIGS solar cells are widely recognized as the most compatible solar cell technology regarding future energy applications [3,4]. Although various deposition processes for CIGS absorbers have been successful and demonstrated a conversion efficiency of approximately 20%, the most successful fabrication processes that surpass 20% efficiency are the three-stage co-evaporation that achieved 22.6%, as proposed by the ZSW group [5], and sulfurization after selenization (SAS) of the CuGaIn metallic precursor (two-step SAS CIGS) (23.35% by Solar Frontier [1]). The CIGS light absorber deposition process strongly affects the microstructural characteristics of films, such as film density, grain size, preferred orientation, surface roughness, and grain boundaries [6]. For instance, the CIGS films produced by three-stage co-evaporation possess a compact large grain size and smooth surface morphology, while those produced by the two-step SAS process exhibit a relatively small grain size and rough surface [7,8].

During the fabrication of the CIGS solar cell, the front contact is usually made of transparent conductive oxide (TCO) materials. The typical TCO front contact must possess low resistivity to enable the transport of electrons and excellent transparency (>80%) to allow the incident solar light to reach the CIGS absorber with minimal absorption loss [9].

The most popular TCO is indium tin oxide (ITO), which has high conductivity, work function, and excellent optical properties, making it suitable for commercial applications [10]. However, the chemical instability and toxicity of indium, as well as its expensive cost due to scarcity, have spurred researchers to look for alternatives to ITO [11,12]. ZnO-based TCO materials, particularly Al-doped ZnO (AZO) thin films, have attracted tremendous interest because of their low cost, natural abundance of constituent elements, outstanding thermal stability, excellent electrical properties, environmental safety, and wide optical band gap (~3.3 eV) [13,14].

AZO films are widely used as front contacts in the device structure of diverse types of solar cells, such as CIGS, CdTe, SnS, Sb_2_S_3_, and a-Si [15,16,17,18,19]. The deposition of AZO thin films for solar cell applications mostly occurs through magnetron sputtering because of its simplicity, high reproducibility, and low cost [20]. In the prior studies of solar cells, the absorber layer and buffer layer (P-N) junction play a significant role in achieving high solar device performance. However, the significance of the AZO layer in device performance is often ignored. As a transparent conductive electrode for solar cells, the AZO conductive layer also plays an essential role in collecting charge carriers and transmitting the solar light to the absorber. Various sputtering parameters such as sputtering power, deposition time, oxygen concentration, and deposition (or substrate) temperature should be studied to improve the electrical and optical properties of the AZO films deposited by sputtering. To limit free carrier absorption, Zhua et al. employed oxygen as a sputtering gas during AZO deposition; nonetheless, the resistivity of the resultant AZO films was increased [21]. Rana et al. showed that increasing the sputtering power from 130 to 160 W reduced the optical bandgap from 3.59 to 3.48 eV [22]. The sample grown at a sputtering power of 160 W had the highest conductivity (2.43 × 10^2^ S m^−^^1^) [22]. Zhou et al. observed improved crystallinity and electrical properties of AZO films with decreasing the sputtering pressure [23]. Patel et al. reported a shift in the band gap to low energy and a decrease in the resistivity as deposition temperature increased from 200 °C to 600 °C [24]. Furthermore, Subramanyam et al. reported a decrease in resistivity from 17.2 to 0.373 mΩ·cm as the substrate temperature increased from room temperature to 300 °C [25].

The above discussion shows that much attention has been paid to studying the effects of sputter-deposition conditions on the properties of AZO films rather than on the overall solar device performance. Only a few studies have investigated the relationship between the AZO deposition conditions and solar cell performance. For example, the impact of sputtering AZO films at room temperature using argon gas with different percentages of oxygen in AZO film properties and on related CIGS solar cell performance was reported by Niu et al. [26]. In their work, the O_2_ concentration was optimized, and the transmittance of the AZO film was improved using 1.5 sccm of the O_2_-Ar gas mixture. This enhanced the short-circuit current density (J_SC_) and overall device efficiency of CIGS solar cells [26]. Sung et al. observed improvements in the CIGS solar cell device parameters and AZO film properties using an oxygen concentration of 0.5% at a substrate temperature of 150 °C [27]. Recently, Zhang et al. correlated AZO properties with the CIGS solar cell performance by varying the AZO deposition power from 250 W to 450 W at room temperature [28]. The electrical and optical properties improved at 350 W, which enhanced the CIGS solar cell efficiency from 14.05 to 15.36% [28]. The device performance of CIGS cells fabricated with two different AZO deposition temperatures (125 and 160 °C) was compared by Chang et al. [29]. They observed that the CIGS device fabricated at the higher AZO deposition temperature (160 °C) showed lower cell efficiency (9.3%) than that (12.1%) prepared at the lower AZO deposition temperature (125 °C), suggesting that the high AZO deposition temperature may affect CIGS/CdS junction quality [29]. However, the effect of AZO deposition temperature on the solar cell performance was not studied in detail.

In this study, CIGS absorbers with different surface textures were prepared by different processes (three-stage co-evaporation and two-step SAS), and the effect of the AZO substrate temperature on the performance of the CIGS solar cells was studied by varying the AZO substrate temperature from 150 to 550 °C.

## 2. Materials and Methods

### 2.1. Thin-Film Deposition

CIGS light absorbers were deposited on glass/Mo substrates by the conventional 3-stage co-evaporation and 2-step SAS processes. During the 3-stage co-evaporation process, In, Ga, and Se were evaporated at a substrate temperature of 400 °C in the first stage, resulting in the formation of the (In,Ga)_2_Se_3_ phase. During the second stage, only Cu and Se elements were supplied at an elevated substrate temperature of 550 °C, resulting in the transformation of (In,Ga)_2_Se_3_ into CIGS, and then CIGS/Cu_x_Se with excess Cu and Se. In the third stage, an additional evaporation of In, Ga, and Se elements reacted with the Cu_x_Se secondary phase on the surface of CIGS, yielding a single-phase CIGS. More details about the conventional 3-stage CIGS co-evaporation process can be easily found in the literature [30]. The other type of CIGS absorber was prepared using the two-step process, where in the first step, the Cu-In-Ga metal precursors were prepared on glass/Mo substrates by magnetron sputtering. In the second step, selenization of the glass/Mo/Cu-In-Ga metal precursors with H_2_Se was performed, followed by sulfurization with H_2_S, to produce Cu(In,Ga)(Se,S)_2_ (also called CIGS here) light absorbers [31].

By utilizing the chemical bath deposition (CBD) technique, cadmium sulfide (CdS) with a thickness of about 60 nm was deposited on the CIGS absorbers, where cadmium sulfate (CdSO_4_) and thiourea ((H_2_N)_2_CS)) were used as Cd^2+^ and S^2−^ ion sources, respectively. The complexing agent was ammonium hydroxide (NH_4_OH). More details of the deposition of CdS films by the CBD method are available in our previous study [32].

Intrinsic ZnO (i-ZnO) films of approximately 100 nm were deposited on the prepared glass/Mo/CIGS/CdS samples via radio frequency (RF) magnetron sputtering using a 3″-diameter i-ZnO disc target. The RF power, base pressure, working pressure, and Ar flow rate were 90 W, 1.6 × 10^−6^ Torr, 8 mTorr, and 30 sccm, respectively. The AZO films were added to the as-prepared glass/Mo/CIGS/CdS/i-ZnO samples and bare soda-lime glass (SLG) by sputtering a high-purity (99.999%) AZO ceramic target (3″-diameter) in a magnetron sputtering system. The RF power was maintained at 150 W, whereas the substrate temperature was varied as 150, 250, 350, 450, and 550 °C. During the RF sputtering process for AZO, the base and working pressures were maintained at 1.6 × 10^−6^ Torr and 3 mTorr, respectively, while the Ar flow rate was 30 sccm. Finally, the deposition of a Ni/Ag layer with a thickness of approximately 1.45 µm by electron beam evaporation, produced a CIGS solar cell with a structure of SLG/Mo/CIGS/CdS/i-ZnO/AZO/Ni:Ag.

### 2.2. Characterization

The SLG/AZO thin films and CIGS absorbers were characterized using several analytical techniques. The crystallographic characteristics were studied by X-ray diffraction (XRD; X’Pert PRO MPD, Malvern Panalytical, Almelo, Netherlands) using Cu-K_α1_ radiation (λ = 1.54056 Å). The CIGS absorber composition was estimated using inductively coupled plasma–atomic emission spectroscopy (ICP-AES; OPTIMA 8300, PerkinElmer, Akron, OH, USA). The surface morphology and thickness of the CIGS absorbers and AZO films were examined using field-emission scanning electron microscopy (FE-SEM; S-4800, Hitachi, Tokyo, Japan). The surface morphology and roughness of the CIGS absorbers and AZO films were investigated using atomic force microscopy (AFM; XE-100, Park System, Suwon, Korea). At room temperature and in the wavelength ranging from 900–300 nm, the SLG/AZO thin films’ optical properties were investigated by the help of double beam UV-VIS-NIR spectroscopy (Cary 5000, Agilent, Santa Clara, CA, USA). The four-probe apparatus (CMT-100S, Advanced Instrument Technology, Suwon, Korea) was utilized to analyze the electrical properties of the deposited SLG/AZO thin film. X-ray photoelectron spectroscopy (XPS; ESCALAB 250, Thermo Fisher Scientific, Altrincham, UK) was employed to investigate the chemical structure of SLG/AZO film. Finally, the current density–voltage (J–V) characteristics of the CIGS solar devices were determined using a solar simulator (K201 LAB 55, McScience, Suwon, Korea) under the one-sun condition, that is, air mass 1.5.

## 3. Results

### 3.1. Composition

The elemental composition of the prepared CIGS light absorbers was estimated by ICP-AES analysis, where the specimen was prepared using a 1 cm × 1 cm piece of the sample dissolved in a HNO_3_ solvent and then diluted with H_2_O. The results are summarized in Table 1. For the three-stage co-evaporated CIGS, the evaluated average ratio of Cu/(In + Ga) and Ga/(In + Ga) is ~0.86 and ~0.24, respectively. For the two-step SAS CIGS, the evaluated average ratio of Cu/(In + Ga) and Ga/(In + Ga) is ~0.86 and ~0.24, respectively. Both CIGS light absorbers prepared using different processes possessed similar atomic ratios, which is consistent with the CIGS composition reported in the literature [33,34,35].

### 3.2. Crystallographic Properties

**Figure 1:**
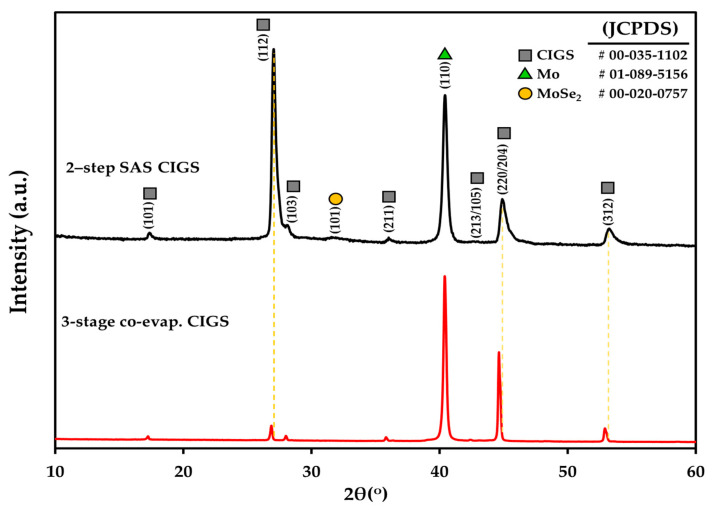
X-ray diffraction patterns of the 2-step SAS CIGS and 3-stage co-evaporated CIGS films deposited on the glass/Mo substrates in this study.

Figure 1 shows the bulk XRD pattern of CIGS thin films grown on SLG/Mo substrates by the two-step SAS and three-stage co-evaporation processes. The preferred orientation of polycrystalline CIGS is usually either (112) or (220). One of these orientations dominates depending on the deposition techniques or conditions [36]. The XRD results in Figure 1 show that both CIGS samples exhibit peaks related to the CIGS compound with a polycrystalline chalcopyrite crystal structure. The two-step SAS CIGS showed an intensity ratio of about 3.73 for the peaks (112)/(220), and this means that (112) is the preferred orientation, while in the case of the three-stage co-evaporated CIGS sample, the intensity ratio of same peaks was about 0.22, which means that (220) is the preferred orientation. It should be noted that the intensity ratio of those peaks is about 2 for the randomly oriented powder CIGS (JCPDF 00-035-1102).

Notably, the preferred orientation of the CIGS light absorber, that is, (112) or (220), plays a critical function in the CIGS absorber characteristics and solar cell device performance, because the variation in its preferred orientation produces different impacts on the grain boundary activities, which influence the transportation of the photoexcited carrier in the CIGS solar cell device [36,37]. The CIGS film with the (112) preferred orientation was reported to have a high photoexcited carrier recombination [38]. Shin et al. found that a CIGS absorber with a (220) preferred orientation was more efficient (16.36%) than the CIGS absorber with the (112) preferred orientation [39]. Londhe et al., who reported the deposition of the CIGS light absorber by electrodeposition [40], observed that the CIGS deposited at −1.6 V had a large grain size with (220) preferred orientation and relatively high efficiency (~9.07%), while that deposited at −0.9 V had a small grain with (112) preferred orientation and lower CIGS device efficiency (~4.90%) [40].

In addition, as shown in Figure 1, the two-step SAS CIGS XRD peaks including (112), (113), and (220/204) were slightly shifted to a greater 2θ angle, and as a result the d-spacings and lattice constants of the CIGS crystal structure reduced due to the incorporation of smaller sulfur atoms [41].

Furthermore, the MoSe_2_ (101) peak was identified in the two-step SAS CIGS sample, but nearly invisible in the three-stage co-evaporated CIGS, which is typically reported in the literature [42].

**Figure 2:**
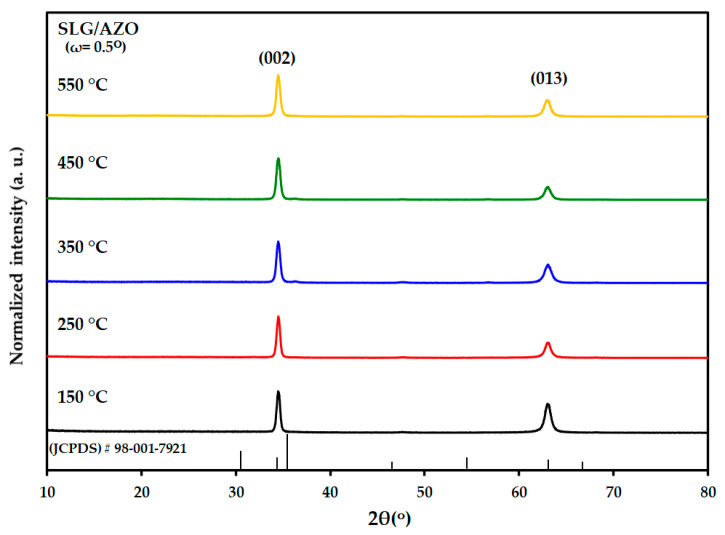
GI–XRD patterns (ω = 0.5°) of the SLG/AZO films prepared with a different deposition temperature.

As shown in Figure 2, the crystallographic properties of the AZO thin films deposited at various substrate deposition temperatures on glass substrates were investigated by glancing incidence XRD (GI–XRD) with an incident angle of ω = 0.5°. GI–XRD was used because of the small thickness in the range of 340–460 nm of the deposited AZO thin films. As shown in Figure 2, the XRD patterns demonstrate that all the AZO films possessed a hexagonal wurtzite structure, regardless of the substrate temperature [43,44]. Furthermore, for all the deposited AZO thin films, only two peaks for ZnO are observed at approximately 34.5° and 63.20° for the (002) and (013) planes, respectively. In addition, a comparison with the XRD powder diffraction (JCPDF. 98-001-7921) database showed that the (002)/(013) peaks’ intensity ratio exceeded 1.45, compared to 1.20 for powder. This further confirmed that the preferred orientation of all the deposited AZO films was (002) [43].

### 3.3. Morphological Properties

**Figure 3:**
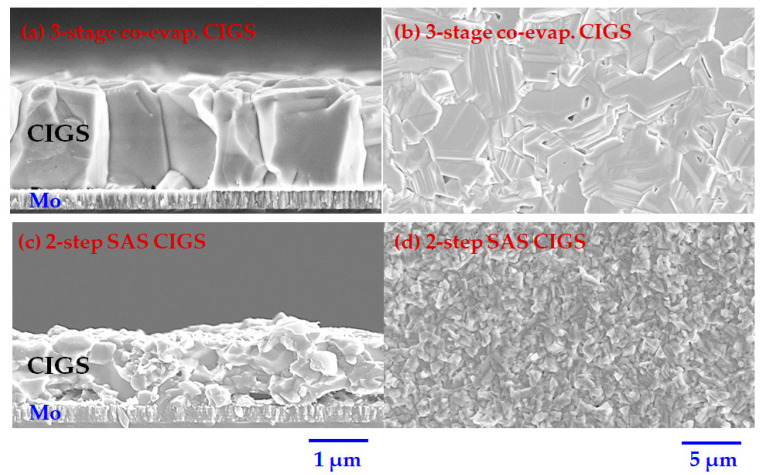
Surface and cross-sectional SEM images of the (**a**,**b**) 3-stage co-evaporated CIGS and (**c**,**d**) 2-step SAS CIGS.

SEM analysis was used to investigate the microstructure of the prepared CIGS absorber using different methods. Figure 3 clearly shows the different microstructures and morphologies of the three-stage co-evaporated CIGS and two-step SAS CIGS. The CIGS light absorber prepared by the three-stage process has a large grain and smooth surface, while the two-step SAS CIGS has a smaller grain with a relatively rough surface. It is well known that during the deposition of the CIGS absorber by the three-stage process, the smooth layer of (In,Ga)_2_Se_3_ formed during the first stage helps in producing a final CIGS layer that is remarkably smoother as compared to that produced by the SAS of metallic precursors in the furnace [45].

**Figure 4:**
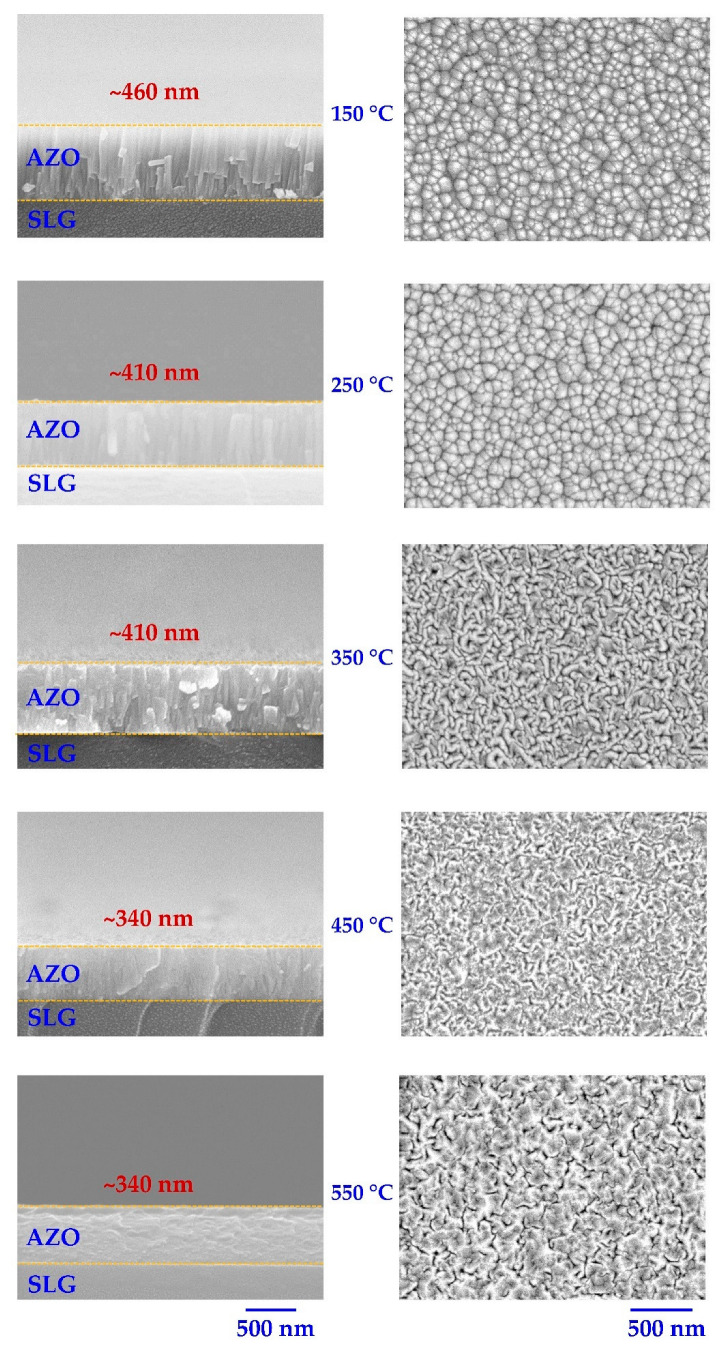
SEM cross-sectional and surface images of SLG/AZO deposited at different substrate temperatures.

As shown in Figure 4, the SEM was used to investigate the impact of substrate temperature on the thickness and surface morphology of sputter-deposited AZO films. Because the AZO films’ surface morphology greatly affects their electrical and optical properties, a detailed analysis of the alteration in morphology for various substrate (or deposition) temperatures is essential. Noticeably, the SEM cross-section of the deposited AZO films with various substrate temperatures indicated a decrease in the film thickness with increasing substrate temperature. Moreover, the cross-sectional and surface SEM images show that at low temperatures of 150–350 °C, the AZO films have a columnar perpendicular grain, whereas at high temperatures of 450–550 °C, these columnar grains are compressed and overlapped, and the AZO films became denser.

The decrease In AZO thickness with increasing substrate temperature can be attributed to two factors. First, at a high deposition temperature, the AZO absorbed atoms diffuse and rearrange themselves to pack the gap between the columnar crystals [46,47,48]. Therefore, the AZO films deposited at higher temperatures are smooth, compact, dense, and exhibit a smaller film thickness [46,47,48]. Second, the high substrate temperature promotes the desorption of the absorbed atoms that possess lower kinetic energies; therefore, the deposited film thickness decreases at high substrate temperatures [46,47,48].

**Figure 5:**
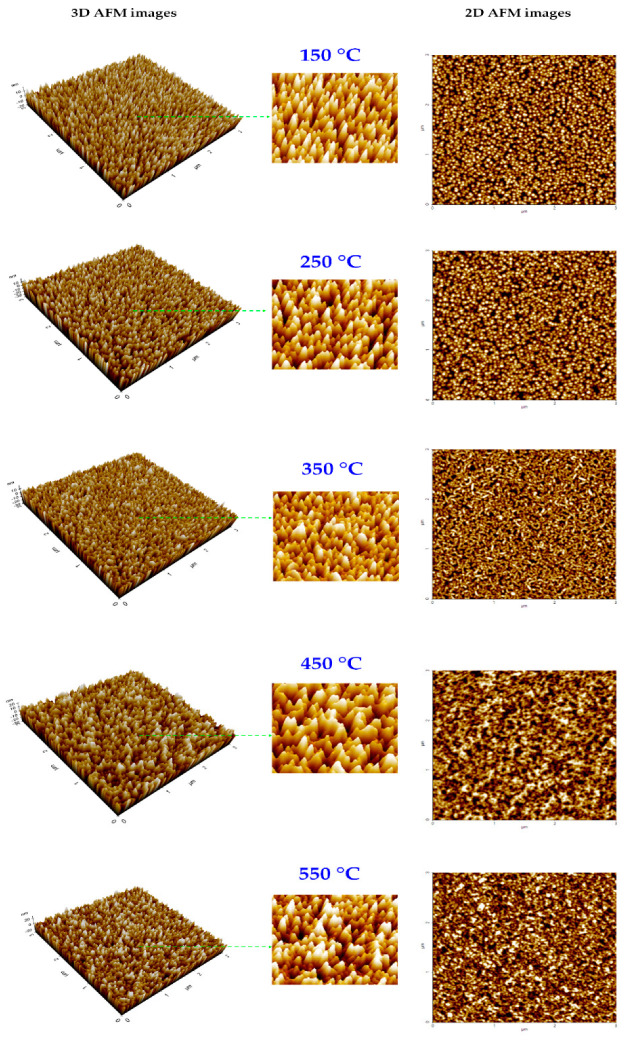
The 3D and 2D AFM images of SLG/AZO thin films deposited at different substrate temperatures.

**Figure 6:**
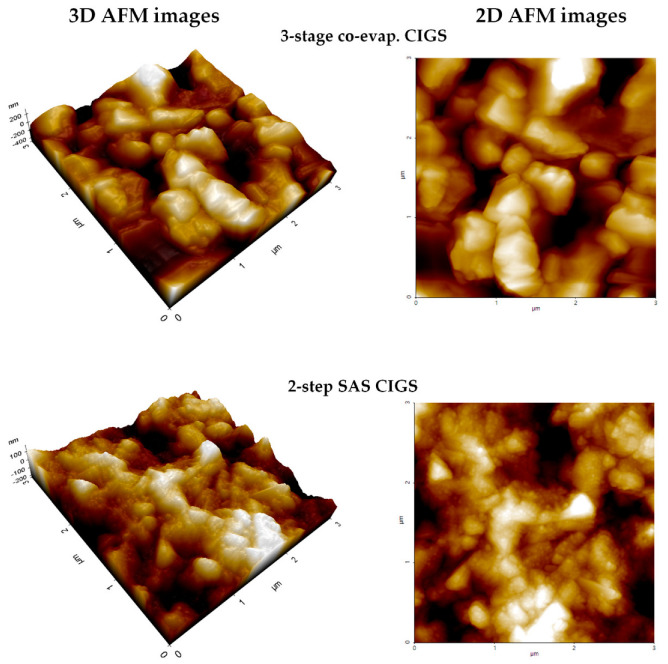
The 3D and 2D AFM images of CIGS absorbers prepared by the 3-stage co-evaporation and 2-step SAS processes.

Figure 5 shows the 3D and 2D AFM images of the AZO thin films deposited at different substrate temperatures. The root mean square (RMS) surface roughness (Rq) of the AZO films was estimated for a surface area of 3 × 3 μm^2^. All the AZO films were observed to possess similar RMS roughness (Rq ~6 nm). However, from the 3D AFM images in Figure 6, it is apparent that at low substrate temperatures (150–250 °C), the AZO films have a columnar grain morphology. As the substrate temperature increased further (350–550 °C), the columnar grains in the AZO films started intermixing, forming larger grains and a flatter surface. The grain growth at high substrate temperatures caused similar film roughness, although the 3D and 2D AFM images displayed a smoother AZO film at high substrate temperatures. The results of the AFM analysis were consistent with those of the SEM analysis.

Figure 6 presents the 3D and 2D AFM images of the CIGS light absorbers grown by the three-stage co-evaporation and two-step SAS processes. The RMS surface roughness (Rq) (surface area of 3 × 3 μm^2^) of the three-stage co-evaporated CIGS absorber is estimated to be approximately 93 nm, which is higher than that of the two-step SAS CIGS absorber (approximately 63 nm). This is predictable because the three-stage co-evaporated CIGS absorber has larger grains compared to the two-step SAS CIGS absorber with small grains.

### 3.4. Optical Properties

UV-Vis-NIR spectroscopy was used to measure the optical properties of the SLG/AZO thin films deposited at different substrate temperatures at wavelengths ranging from 300 to 900 nm.

**Figure 7:**
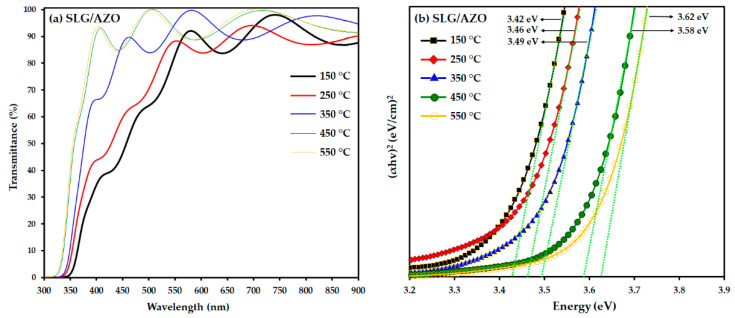
Optical properties of the SLG/AZO thin film analyzed by UV-VIS spectroscopy: (**a**) transmittance and (**b**) optical band gap.

Figure 7a shows that the AZO films’ transmittance improved as the substrate temperature increased. The enhancement in AZO transmittance with the increase in the deposition temperature of the AZO film can be related to the improvement in the grain sizes and reduction in the thickness of the AZO films with the increase in the AZO deposition temperature. In particular, the grain size has a significant impact on the optical properties of thin films, because large grain sizes reduce light scattering at the grain boundaries [49,50,51].

Further, Figure 7b shows that the AZO thin films’ band gap energy shifted to a higher energy (from approximately 3.42 eV to approximately 3.62 eV) as the substrate temperature increased from 150 to 550 °C. This behavior can be associated with the increase in the carrier concentration of the AZO films with increasing substrate temperature. The increase in the band gap energy with increasing substrate temperature is well known as the Burstein–Moss effect, in which the band gap of the semiconductor widens as the absorption edge is shifted to higher energies by filling some states near the conduction band [52]. Therefore, an increase in carrier concentration with increasing substrate temperatures supported the diffusion of additional Al atoms into the ZnO and partially replaced the Zn^2+^ ions at high substrate temperatures [52]. Similar results were reported previously [53,54]. These results are also consistent with the conductivity results of AZO films obtained from four-point probe measurements in the following section.

**Figure 8:**
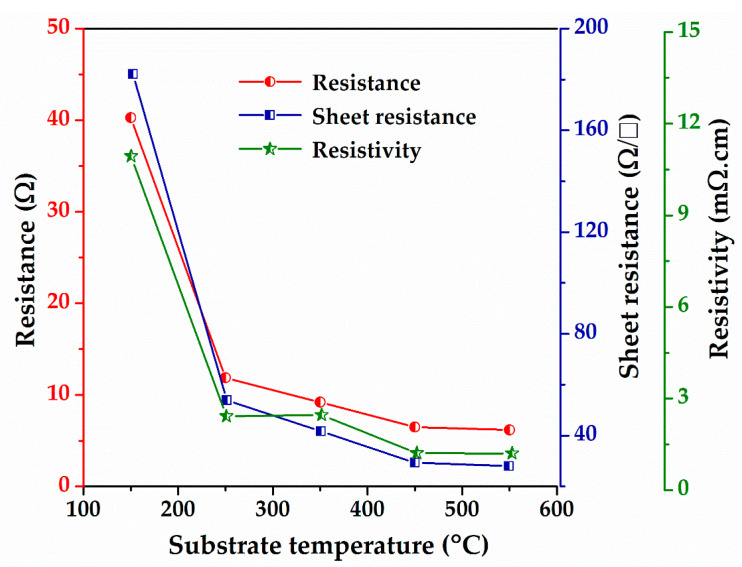
Four-point probe results of the SLG/AZO thin films as a function of substrate temperature.

Figure 7 shows the four-point probe measurement results of the AZO films as a function of the substrate temperature. As shown in Figure 8, the resistance, sheet resistance, and resistivity of the AZO films decreased dramatically with increasing substrate temperature. The resistance of the AZO film deposited at 150 °C was approximately 40.28 Ω, but it decreased rapidly to ~11.87 Ω when the substrate temperature was increased to 250 °C. In addition, the resistance decreased monotonically with increasing the substrate temperature to 550 °C, while the resistance nearly saturated at 450 °C and higher temperatures. Following a similar trend, the sheet resistance and resistivity decreased exponentially from 182.10 to 28.03 Ω/□ and from 10.94 to 1.20 mΩ·cm, respectively, as the substrate temperature changed from 150 to 550 °C. These results indicate that the conductivity of the AZO films increased with the substrate temperature, as reported in a previous study [55]. Park et al. reported a decrease in the resistivity of the AZO film from 8.046 × 10^−4^ to 1.297 × 10^−4^ Ω·cm as the substrate temperature increased from room temperature to 500 °C [55]. They concluded that the improvement in the electrical properties of AZO films is related to the improved crystallinity and reduced grain boundary scattering with increasing substrate temperature [55].

### 3.5. Chemical States

**Figure 9:**
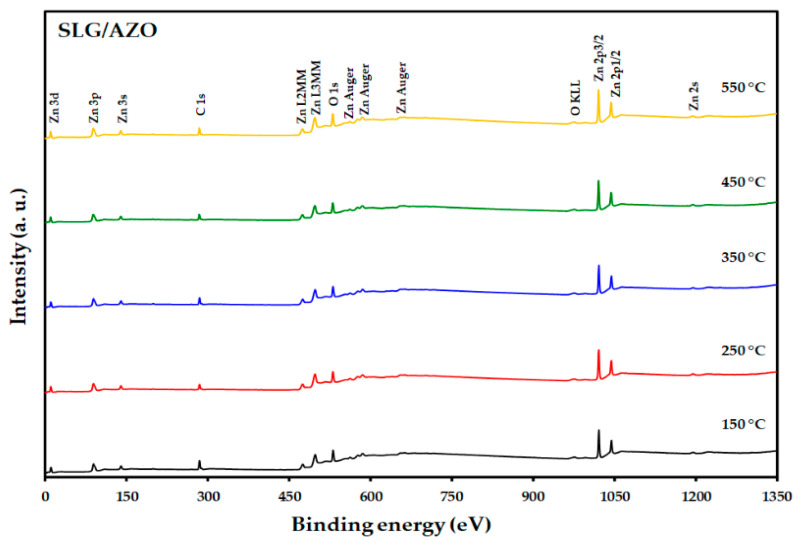
XPS survey spectra of the SLG/AZO thin films as a function of the substrate temperature.

The XPS analysis technique was adopted to investigate the chemical state of the prepared AZO films. The XPS wide scan of the SLG/AZO films demonstrated the presence of the Zn 2p, O 1s, and Al 2p energy regions, as shown in Figure 9, with a weak peak corresponding to C 1s observed at about 285.08 eV. This corresponds to the C-C bond, which could appear owing to contamination (e.g., CO_2_, CO) at the film surfaces from the surrounding atmosphere [56]. The C 1s peak served as a reference for calibrating the binding energies of other elements.

**Figure 10:**
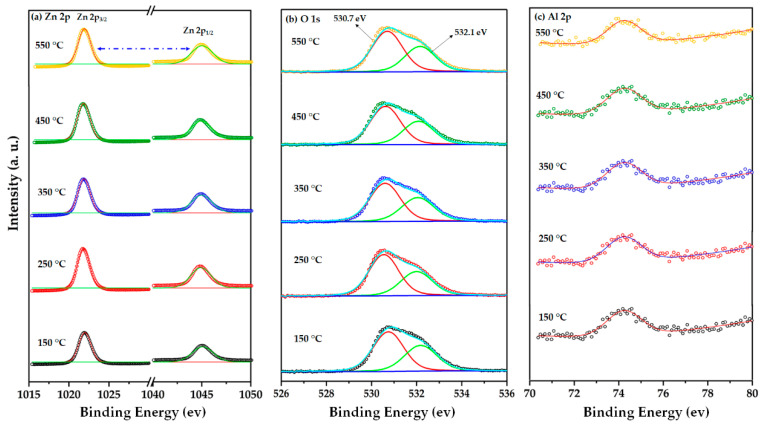
High-resolution XPS spectra for the (**a**) Zn 2p, (**b**) O 1s, and (**c**) Al 2p of the SLG/AZO thin films as a function of the substrate temperature.

The high-resolution XPS spectra of Zn 2p (Figure 10a) shows doublet peaks at about 1021.3 and 1044.7 eV that related to Zn 2p_3/2_ and Zn 2p_1/2_, respectively, with the absence of a significant peak shifting with a change in substrate temperature during AZO sputtering [57]. The appearance of Zn 2p peaks in this position confirmed the ^+^2 oxidation state of Zn for the ZnO phase [57]. Further, the binding energies of these two peaks differed by approximately 23.4 eV, which also suggests that Zn has a ^+^2 oxidation state for the ZnO phase [57]. Figure 10b shows the high-resolution XPS spectra of O 1s, which could be decomposed into two Gaussian peaks at binding energies of 530.7 and 532.1 eV. The O 1s peak observed at the lower binding energy (530.7 eV) is associated with the oxygen lattice in the Zn–O hexagonal structure, which originated from the tetrahedrally coordinated Zn^2+^ and O^2−^ ions [58,59,60]. However, the O 1s peak located at greater binding energy (532.1 eV) corresponds to the regions deficient of oxygen in the ZnO structure [58,59,60]. Figure 10c shows that the Al 2p binding energy is approximately 74.02 eV for deposition temperatures between 150–550 °C. Based on Gaussian fitting, the Al 2p component located at approximately 74.02 eV is a typical characteristic of Al for Al_2_O_3_ [61]. The XPS spectra of Al 2p confirm the total oxidation of Al, which is supported by the absence of the metallic Al 2p XPS peak at a binding energy of ~74 eV [61]. Therefore, the XPS analysis indicates that the prepared AZO films possess similar chemical structures, and no significant peak shifting occurs with the deposition temperature.

### 3.6. Device Performance

**Figure 11:**
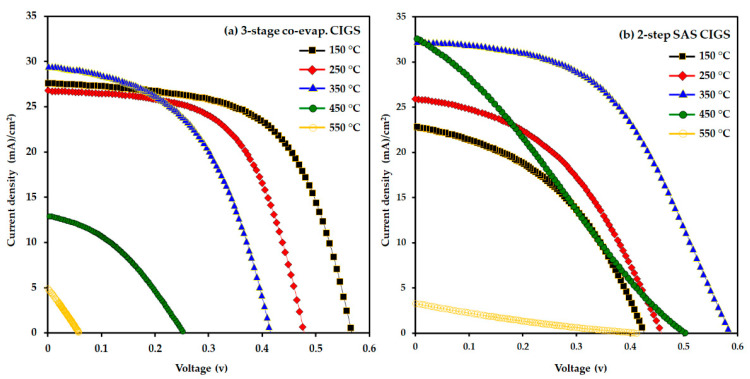
J–V curves of (**a**) 3-stage co-evaporation and (**b**) 2-step SAS-processed CIGS solar cells as a function of the AZO substrate temperature.

**Figure 12:**
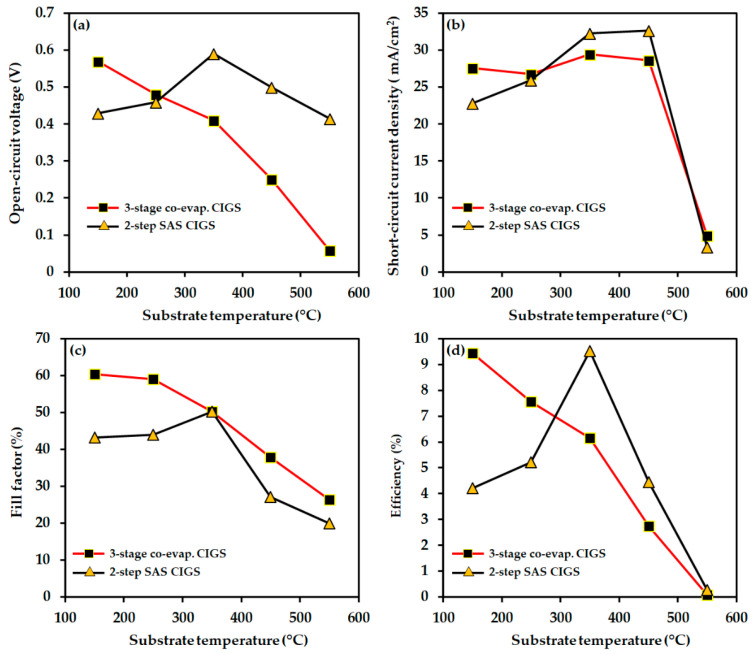
Solar cell device parameters as a function of the AZO layer substrate temperature: (**a**) open-circuit voltage (V_OC_), (**b**) short-circuit current density (J_SC_), (**c**) fill factor (FF), and (**d**) efficiency (η).

The J–V characteristics and device performance parameters of the as-fabricated solar cells (with the structure mentioned in the experimental section) using the three-stage co-evaporated and two-step SAS-processed CIGS light absorbers as a function of AZO substrate temperature are shown in Figure 11 and Figure 12, while the photovoltaic device parameters are summarized in Table 2. The results demonstrate that the change in the AZO substrate temperature has a significant impact on the CIGS cells performance.

In the case of the three-stage co-evaporated CIGS cells, the cell efficiency decreased significantly from 9.43 to 2.75% as the AZO substrate temperature increased from 150 °C to 450 °C, and ultimately deteriorated at 550 °C. Concurrently, the open-circuit voltage (V_OC_) and fill factor (FF) values decreased, but the short-circuit current density (J_SC_) value improved slightly from 27.57 to 29.46 mA/cm^2^ up to 350 °C and then deteriorated. It was deduced that the improvement in the transmittance and conductivity of the AZO layer with increasing substrate temperature of AZO (from 150 to 350 °C) played a critical role in improving J_SC_ [62]. However, in the case of two-step SAS-processed CIGS devices, the device efficiencies improved almost twice from 4.21 to 9.51% as the AZO substrate temperature increased from 150 to 350 °C, which was benefited from improving the other device parameters (e.g., J_SC_ and FF). However, upon further increasing the AZO substrate temperature, the device parameters decreased and eventually degraded at 550 °C, similar to the three-stage co-evaporated CIGS cells. Therefore, AZO substrate temperatures of 150 °C and 350 °C are considered as the optimum values for the three-stage co-evaporated and two-step SAS-processed CIGS cells, respectively.

The above results indicate that the optimal AZO substrate temperature depends on the CIGS absorber deposition process, and the three-stage co-evaporated CIGS requires a low optimal AZO substrate temperature compared to the two-step SAS-processed CIGS. This could be because the different processes produced CIGS absorbers with different preferred orientations and different degrees of diffusion of Cd into CIGS absorbers during the CdS buffer layer deposition or from the CdS buffer layer during TCO deposition. The incorporation of several buffer material elements such as Cd from CdS and Zn from ZnS into the CIGS absorber and their impact on device performance have been reported previously. Cho et al. [63] varied the ITO deposition temperature from room temperature to 350 °C and found that the CIGS device performance improved when ITO was deposited at temperatures ≤200 °C; at >200 °C, device performance began to deteriorate. Additionally, secondary ion mass spectrometry (SIMS) results proved that the diffusion of Cd from CdS into the CIGS absorber widened the space charge region (SCR), which increased the recombination rate and destroyed the CIGS device performance [63,64,65,66]. Nakada et al. [67] observed that after the deposition of the CdS buffer layer, the presence of Cd in the CIGS layer was approximately 10 nm, along with a decrease in the Cu concentration at the surface of the CIGS absorber from the interface boundary. Furthermore, they concluded that Cd diffusion into CIGS occurred rapidly, owing to Cu deficiency. Koprek et al. [65] annealed the CIGS device and characterized Cd diffusion inside a CIGS absorber using the atom probe tomography (APT) technique. Their results indicated that a remarkable amount of Cd was incorporated into the CIGS absorber when the sample was annealed above 150 °C. Further, in films prepared at temperatures over 200 °C, the elemental redistribution analysis associated with capacitance–voltage (C–V) measurement confirmed the existence of CdCu+ donor defects deep within the CIGS. This resulted in an intense compensation of the CIGS absorber and significant deterioration of the CIGS device performance. Lee et al. [68] observed an improvement in the minority carrier lifetime and CIGS device performance owing to the migration of a small amount of Zn from the (Zn, Mg)O buffer into CIGS when the entire device was annealed at 200 °C, whereas a significant amount of Zn diffused into the CIGS absorber and reduced the device efficiency and minority carrier lifetime when the samples were annealed above 200 °C. Lee et al. [68] concur with Nakada et al. [69], who concluded that the diffusion of trace amounts of Cd into the CIGS absorber could produce n-type inversion behavior at the CIGS surface via Cd–Cu substitution, yielding a better p–n junction and enhanced CIGS device performance.

According to the CIGS device results presented here, the performance of the three-stage co-evaporated CIGS device degraded rapidly with increasing AZO substrate temperature, whereas the cell performance improved up to 350 °C in the case of the two-step SAS-processed CIGS devices. The other possible reason for the different cell performance behaviors with substrate temperature is the preferred orientation of the CIGS absorber, as mentioned earlier, which is considered to play an essential role in controlling the diffusion of impurities (e.g., Cd, Zn) through the CIGS absorber. Chaisitsak et al. [37] reported two types of CIGS absorbers with preferred orientations of (112) and (220) and compared their Cd diffusion behaviors. Their results showed that Cd atoms diffused more rapidly through the (220) plane-oriented CIGS absorber than through the (112) plane-oriented films [37]. In addition, they concluded that Cd diffused more easily into the CIGS absorber with (220) preferred orientation compared to the absorber with (112) preferred orientation because of the comparatively lower atom density of the (220) plane [37]. As shown in Figure 1, the three-stage co-evaporated CIGS absorber had a (220) preferred orientation, which favors the diffusion of Cd atoms. It explains the rapid degradation in the CIGS device performance caused by a large amount of Cd diffusion into the CIGS absorber with increasing AZO substrate temperature. In contrast, the diffusion of Cd atoms into the two-step SAS-processed CIGS absorber with (112) preferred orientation is more difficult, which results in the improved performance of the CIGS devices with increasing AZO substrate temperature.

Therefore, it is concluded that the texture of the CIGS absorber plays a significant role in determining the optimum AZO substrate temperature. Although the AZO layer properties improved greatly with the substrate temperature, the CIGS with different grain orientations responded differently. Thus, the deposition temperature for the TCO layer for heterojunction thin-film solar cells must be carefully optimized.

## 4. Conclusions

In this study, different CIGS light absorbers with various surface roughnesses, preferred crystal orientations, and morphologies were fabricated by the two-step SAS and three-stage co-evaporation processes. The CIGS light absorbers with different characteristics required different optimum AZO substrate (or deposition) temperatures. For the solar cell devices fabricated with three-stage co-evaporated CIGS light absorbers, the optimum AZO layer substrate temperature was determined to be 150 °C. With further increase in the AZO substrate temperature, the solar device performance broke down regardless of the improvement in the AZO layer with increasing temperature. For the solar cell devices fabricated using two-step SAS CIGS light absorbers, the optimum AZO layer substrate temperature was determined to be 350 °C, which benefited from the improvement of AZO layer properties with increasing substrate temperature. This study investigated the strong correlation between the crystal orientation of the CIGS absorbers and Cd doping from the CdS buffer layer into the CIGS absorber, where we observed that Cd could be doped rapidly into three-stage co-evaporated CIGS with (220) preferred crystal orientations, as indicated by the rapid deterioration of solar devices with increasing AZO deposition temperature. Conversely, the slow Cd doping into the (110) plane-oriented two-step SAS CIGS absorbers improved their solar cell devices by enhancing the AZO layer properties with increasing substrate temperatures. Hence, we conclude that the deposition temperature for the TCO layer in heterojunction thin-film solar cells should be carefully optimized.

## Figures and Tables

**Table 1:** Composition of the CIGS light absorbers estimated using ICP-AES.

CIGS Process	Atomic Composition				Atomic Ratios	
	Cu	In	Ga	Se	Cu/III	Ga/III
3-Stage co-evaporation	0.22	0.19	0.06	0.53	0.86	0.24
2-Step SAS	0.26	0.2	0.07	0.46	0.93	0.25

**Table 2:** Performance of CIGS solar cells fabricated using 3-stage co-evaporated and 2–step SAS- processed CIGS absorbers as a function of the AZO substrate temperature.

Growth Process of CIGS	AZO Substrate Temperature (°C)	Solar Cell Performance Parameters			
		J_SC_ (mA/cm^2^)	V_OC_ (V)	FF (%)	Efficiency (%)
**3-stage co-evaporation**	150	27.57	0.57	60.33	**9.43**
	250	26.77	0.48	59.04	7.55
	350	29.46	0.41	50.20	6.14
	450	28.62	0.25	37.87	2.75
	550	4.85	0.057	26.29	0.07
**2-step SAS**	150	22.85	0.43	43.23	4.21
	250	25.92	0.46	43.90	5.22
	350	32.28	0.59	50.15	**9.51**
	450	32.67	0.50	27.07	4.45
	550	3.30	0.414	19.92	0.27

## Data Availability

Not applicable.
